# The experience of loneliness among young people with depression: a qualitative meta-synthesis of the literature

**DOI:** 10.1186/s12888-020-02818-3

**Published:** 2020-08-24

**Authors:** Louis Achterbergh, Alexandra Pitman, Mary Birken, Eiluned Pearce, Herman Sno, Sonia Johnson

**Affiliations:** 1 UCL Division of Psychiatry, Maple House, 149 Tottenham Court Road, London, W1T 7NF UK; 2 Amsterdam UMC, location AMC (University of Amsterdam), Meibergdreef 9, 1105 Amsterdam, AZ Netherlands; 3 Camden and Islington NHS Foundation Trust, St Pancras Hospital, 4 St Pancras Way, London, NW1 0PE UK

**Keywords:** Loneliness, Social isolation, Depression, Meta-synthesis, Systematic review, Qualitative research

## Abstract

**Background:**

Young people have a higher prevalence of loneliness than other age groups, and they are also at risk of depression. Quantitative studies describe a bidirectional association between loneliness and depression, but there is limited understanding of how these influence each other. Little is known about the experience of loneliness among young people with depression. Qualitative approaches may help understand the relationship between loneliness and depression among young people, and how to intervene to improve outcomes. We aimed to conduct a meta-synthesis to understand the complex inter-relationship between loneliness and depression among young depressed people by synthesising evidence from a systematic review of qualitative studies.

**Methods:**

We conducted a meta-synthesis of qualitative studies capturing experiences of loneliness among young people with depression. We systematically searched six electronic databases for selected search terms, critically appraised eligible studies, and analysed the data from included studies using the approach of thematic synthesis. We used feedback from an inter-disciplinary research workshop to improve reflexivity.

**Results:**

Our inclusion criteria identified fourteen studies. Our analysis identified four themes: (1) social withdrawal due to poor mental health, (2) non-disclosure of depression contributing to social distance, (3) the desire to connect, and (4) paradoxes of loneliness and depression. These themes illustrated a range of pathways between depression and loneliness, and a sense of how these might be mutually reinforcing. Our findings suggest that where depressed individuals engage in certain behaviours (withdrawing; not confiding) for a range of reasons, this can lead to feelings of loneliness, an awareness of which worsens their mood, thus perpetuating their depression.

**Conclusions:**

Young people with depression experience loneliness as an insurmountable distance between themselves and others. Our findings identified non-disclosure of depression, and the debilitating nature of the depressive symptomatology, as factors perpetuating a vicious cycle of loneliness and depression. They suggest that approaches to tackling the problem might include helping young people communicate about their depression to trusted friends and educating their social networks in how to support them. The wider research literature suggests that cognitive interventions may have a role in shifting maladaptive cognitions about their social world.

## Background

Loneliness is defined as a negative emotional state that arises when there is a perceived discrepancy between desired and actual social relationships [1]. The adverse effects of loneliness on mental [2], and physical health [3] are now well established. Most work describing the association between loneliness and mental illness has focussed on depression. Depression is the third leading cause of disease worldwide [4], based on World Health Organisation (WHO) rankings, and its incidence appears to be increasing internationally [5]. Onset of major depression extends from mid-adolescence to the mid-40s, but almost 40% experience their first episode of depression before age 20 years, with peaks in prevalence in the second and third decades of life [4]. Cross-sectional work shows that people with depression are ten times more likely to feel lonely than the general population [6]. Longitudinal studies demonstrate that loneliness not only increases the risk of becoming depressed [7–9], and worsens depressive symptoms amongst those who are already depressed [10], but also that loneliness and depression influence each other reciprocally [11]. This means that people who are lonely are more likely to be become depressed, but also that their depression reinforces their loneliness. The mechanisms underlying this complex inter-relationship between loneliness and depression are unclear and need further investigation, particularly in young people.

Population-based surveys describe a U-shaped age distribution of loneliness, with high rates of loneliness among young people and among the elderly [12]. However, the majority of epidemiological work on the health impacts of loneliness have been conducted in older age groups [7–9, 13, 14]. Extrapolating the findings of studies in older age groups to younger people is problematic given that experiences of loneliness vary in different demographic and cultural groups [15]. The social context of loneliness is also very different in young people to later stages of life. Additionally, the experience of depression is also likely to vary by age, with symptoms of irritability and interpersonal difficulties being particularly prominent among adolescents [16].

The few studies that have been done in young people suggest that social isolation in childhood predicts loneliness in young adulthood [17] and that chronic peer-related loneliness in childhood predicts adolescent depression [18–20]. Given the evidence from studies of older age groups, loneliness is likely to be a factor affecting quality of life and prognosis among young people with depression. It is also likely to compound the barriers described by young people in accessing formal or informal help, namely the stigma of mental illness and a reluctance to talk about feelings or emotions [21]. Adolescence and young adulthood is the greatest risk period for the emergence of depression and also one in which loneliness might be most stigmatising given strong social pressure to appear connected [22]. In view of the high prevalence of loneliness amongst young people, and the lack of research focussed on this age group, it is important to gain a better understanding of the experience of loneliness among young people with depression, as well as its causes and consequences, to tailor the design of acceptable age-appropriate treatments [10].

Loneliness is a subjective construct related to the concepts of social isolation [23], alienation [24], social connectedness [25], lack of belonging [26] and social capital [23]. Loneliness is distinct from social isolation, which is an objective measure of the absence of relationships with other people [27]. Loneliness is also distinct from solitude in that loneliness is an unpleasant experience, whereas solitude implies a desire to be alone and is not necessarily a negative experience [27]. Quantitative work shows that loneliness and social isolation are moderately correlated and both are associated with depression [28, 29]. Behavioural genetic analysis finds that young people who are lonely are often depressed, partly because the same genes influence loneliness and depression [28]. Environmental factors are also important; lonely young adults are more likely to have been bullied and socially isolated as children [17]. The distinction between loneliness and social isolation is important because socially isolated young adults do not necessarily feel lonely [28] and young adults who feel lonely do not necessarily spend less time with others in comparison to their less lonely peers [30].

Available evidence suggests that different age groups experience loneliness differently. Comparison of the social networks of young and middle-aged adults show that young adults reported twice as many days feeling lonely and isolated than late middle-age adults, despite having larger networks [31]. Interview data from an English community sample show that children and young people aged 10 to 24 years describe loneliness as a sense of exclusion, disconnection from others and unhappiness with relationships [32]. Children as young as 5 years understand a concept of loneliness, a sadness associated with this, and how it motivates them to make contact with others [19].

By understanding how loneliness and depression influence each other in young people, there is potential for improving depressive symptoms and depressive outcomes through well-developed and appropriately targeted interventions focused on loneliness. The aim of this meta-synthesis was therefore to summarise qualitative research describing the experience of loneliness and depression among young people, to provide insights into the relationships and pathways between them.

## Methods

### Design

Meta-synthesis is a research method that uses rigorous qualitative methods to synthesize existing qualitative studies, with the aim of constructing greater meaning through an overarching interpretation based on the qualitative studies included [33–36]. Thematic synthesis is influenced by the meta-ethnography process and grounded theory [35] and involves conceptual coding of data to construct an encompassing model providing insights to the phenomenon studied [37]. The approach identifies patterns across qualitative data and aims to enrich the understanding of a topic, creating new theoretical insights as well as serving as a tool to develop suitable interventions [38]. It entails an iterative cyclical process comparing and contrasting themes between different studies and attempting to encompass the data using a set of themes that are relevant within each study, constructed as a hierarchical tree structure [35].

For this study we applied an accepted six step method for conducting a meta-synthesis [35], consisting of: 1) defining the research question and selection criteria, 2) using those criteria to select studies, 3) undertaking a quality assessment, 4) extracting and presenting formal data, 5) conducting data analysis and 6) reporting the synthesis.

### Protocol and search strategy

Before commencing, we registered our meta-synthesis protocol with PROSPERO, the international prospective register for systematic reviews [39]. We conducted our search using six electronic databases (MEDLINE, PsycINFO, CINAHL, Scopus, ProQuest and Web of Science) from database inception to 21 March 2019. These databases were chosen to capture studies from a range of research disciplines, including medicine, psychology, sociology and anthropology. We developed search terms as a team (see Appendix 1), including terms capturing depression and mental ill-health, loneliness and social isolation, and qualitative research. Given the conceptual overlap between loneliness and other constructs such as perceived social support, our search terms also included several other words capturing these concepts [10]. We also conducted a search of the Ethos British Library database to find any relevant PhD dissertations and hand searched the reference lists of any eligible studies to reduce the chance of missing important studies.

### Selection: inclusion & exclusion criteria

We screened titles and abstracts of identified studies for eligibility, followed by full text review where indicated, using the following inclusion criteria to identify studies that:

- used a qualitative research design such as semi-structured interviews or focus groups.
- sampled adolescents and/or young adults aged 11 to 30 years with a depressive disorder. We chose the age range 11 to 30 years to cover WHO definitions of adolescents (aged 10–19 years), youth (aged 15–24 years), teenagers (aged 15–19 years), and young adults (aged 20–24 years), with a wider margin at the upper limit in order to ensure that we did not exclude studies including young adults as a proportion of those sampled or studies where young people reflected back on their recent adolescence.
- explored how young people with depression experience loneliness, both in relation to a current depressive episode and/or in reflecting back on past episodes
- included participants with self-reported depression or depression diagnosed by a health professional, regardless of severity of depression or treatment received. We included studies that involved participants with depression, with or without a comorbid anxiety or personality disorder. We also included studies in which the depressive episode was in the context of a diagnosis of bipolar disorder.

Studies were excluded if they:

- sampled participants above the age of 30 only, or used a mixed sample of age groups above and below 30
- sampled participants without a history of depression.
- sampled participants with: a co-morbid chronic physical disability (e.g. rheumatoid arthritis); any co-morbid mental illness other than an anxiety disorder or personality disorder; or a co-morbid neurocognitive disorder (e.g. Alzheimer’s disease). This was to avoid capturing the experience of depression in the context of co-morbid conditions beyond common mental disorders.
- presented data mentioning loneliness fleetingly or not at all. For example, a study with data on one participant saying: ‘I feel lonely because I’m depressed’ would not be deemed sufficient in detail to convey anything meaningful about the experience of loneliness.
- presented data describing solely the objective presence or absence of social support, rather than subjective feelings about perceived social support, social isolation, or social network size.
- used a quantitative research design.
- were not written in English or Dutch.

### Data screening and extraction

One researcher (LA) conducted the search, removed duplicates, and screened the titles and abstracts of all studies for relevance, before assessing the full text of identified studies for eligibility. Three researchers (MB, EP and AP) were each randomly assigned 10% of these studies for full text assessment to ascertain agreement over inclusion/exclusion, meeting regularly as a group to discuss eligibility.

### Quality appraisal

One researcher (LA) appraised all eligible studies for quality using the Critical Appraisal Skills Programme (CASP), a 10-item quality assessment tool for qualitative research [40], discussing this with the wider team. Studies were appraised on these items grouped under three categories; validity (clarity of research aims, appropriateness of qualitative methodology, research design, recruitment strategy, and data collection, appropriate consideration of researcher reflexivity), results (ethical considerations, appropriateness of data analysis, clarity of findings stated), and utility (the value of the research). Study characteristics and appraisal criteria were summarised in a proforma (Table 1). We chose not to exclude studies based on our assessment of low quality. Instead, our synthesis of findings took into account our CASP-based judgements on the quality of included studies, as suggested in methodological guidance [35, 38], and can therefore be interpreted in this context.

**Table 1:** Table presenting characteristics and quality appraisal of included studies

Citation	Sample size	Population studied	Country	Aims	Data collection	Analysis	Themes	Quality Appraisal 1–10
Al-Khattab et al. 2016 [41]	27 (15 ♂)	Aged 18–21. African-American (AA) adolescents having a) depressive symptoms during adolescence (aged 13–17). 22 participants in total or b) currently aged 13–17 and receiving treatment for depression. 5 participants in total	USA	How AA adolescents describe symptoms of depression through relationships with people in their lives.	Semi-structured interviews	Thematic analysis	1) keeping others at bay 2) striking out at others 3) seeking help from others 4) joining in with others 5) having others reach out	7/10 Validity ✓ Clear aims ✓ Appropriate qualitative methodology ✓ Appropriate research design x Appropriate recruitment strategy x Appropriate data collection ✓ Considered reflexivity appropriately Results x Ethical considerations addressed ✓ Rigorous data analysis ✓ Clear statement of findings Utility of results ✓ Value of research
Anttila et al. 2015 [42]	70 (54 ♂)	Aged 15–17 diagnosed with depression (outpatient) without SMI taking part in RCT for internet-based support system	Finland	Adolescent concerns and hopes when referred to outpatient treatment	Written text/essay before intervention	Thematic analysis	1) Relationships 2) Daily actions 3) Identity 4) Well-being	7/10 Validity ✓ Clear aims ✓ Appropriate qualitative methodology x Appropriate research design ✓ Appropriate recruitment strategy x Appropriate data collection x Considered reflexivity appropriately Results ✓ Ethical considerations addressed ✓ Rigorous data analysis ✓ Clear statement of findings Utility of results ✓ Value of research
De Mol et al. 2018 [24]	15 (9 ♂)	Hospitalized (> 4 months) adolescents (aged 15–18) diagnosed with depression by psychiatrist after receiving outpatient psychotherapy	Belgium	The role of social representations in adolescents’ construction of major depression	Semi-structured interviews	Interpretative Phenomenological Analysis	1) Depression means personal failure 2) Feeling bad is not allowed and is not normal: in fact, depression doesn’t really exist 3) You are obliged to have an intimate relationship, otherwise you are not normal; 4) It is important to have future projects for personal and social well-being 5) Being socially well integrated is normality.	10/10 Validity ✓ Clear aims ✓ Appropriate qualitative methodology ✓ Appropriate research design ✓ Appropriate recruitment strategy ✓ Appropriate data collection ✓ Considered reflexivity appropriately Results ✓ Ethical considerations addressed ✓ Rigorous data analysis ✓ Clear statement of findings Utility of results ✓ Value of research
Dundon 2006 [43] (metasynthesis)	107 (94 ♂)	Aged 13–22. 72 diagnosed or self-reported as depression. 6 studies in total, 2 of which unpublished	USA/ Canada	Contribute to the theoretic base of the experience of adolescent depression, affect future research, and guide clinical practice.	Self-reports, semi-structured interviews	TA, Descriptive, narrative, Participatory action, discourse analysis, grounded research	1) Beyond the blues 2) Spiraling down and within 3) Breaking points 4) Seeing and being seen 5) Seeking solutions 6) Taking control.	5/10 Validity ✓ Clear aims ✓ Appropriate qualitative methodology ✓ Appropriate research design x Appropriate recruitment strategy x Appropriate data collection x Considered reflexivity appropriately Results ✓ Ethical considerations addressed x Rigorous data analysis x Clear statement of findings Utility of results ✓ Value of research
Farmer 2002 [44]	5 (3 ♂)	5 adolescents (aged 13–18) diagnosed with depression by therapist.	USA	Experience of major depression from the adolescent’s perspective to provide a more comprehensive description of the disorder.	Semi-structured interviews	Phenomenological approach	1) Dispirited weariness 2) Emotional homelessness (sense of aloneness) 3) Emotional homelessness (no safety where expected) 4) Unrelenting anger 5) Parental break-up: caught in the middle 6) Spectrum of escape from pain 7) Perspectives on friendship 8) Gaining a sense of getting well	9/10 Validity ✓ Clear aims ✓ Appropriate qualitative methodology ✓ Appropriate research design ✓ Appropriate recruitment strategy ✓ Appropriate data collection ✓ Considered reflexivity appropriately Results ✓ Ethical considerations addressed x Rigorous data analysis ✓ Clear statement of findings Utility of results X Value of research
Granek 2006 [45]	6 (1 ♂)	Students (aged 25–30) gone through an episode of clinical depression (meeting DSM 4 criteria) referring to that period. Didn’t meet criteria for current depression.	Canada	Depressive experience from a subjective perspective	Open ended interviews	Grounded theory/hermeneutic approach	1) Self in relation 2) Self-criticism and self-loathing 3) Loneliness and disconnection	6/10 Validity ✓ Clear aims ✓ Appropriate qualitative methodology ✓ Appropriate research design x Appropriate recruitment strategy ✓ Appropriate data collection ✓ Considered reflexivity appropriately Results x Ethical considerations addressed x Rigorous data analysis ✓ Clear statement of findings Utility of results xValue of research
Kuwabara et al. 2007 [46]	15 (10 ♂)	Community sample (18–25) currently experiencing depression determined by physician interviewers (severe depression with suicidality was excluded)	USA	Obtain a relatively unconstrained description of the ways in which depression is construed and experienced among a sample of emerging adults.	Semi-structured interviews	Thematic Analysis	1) Identification as an individual with depression 2) Interactions with the healthcare system 3) Relationships with friends and family 4) Role transitions from childhood to adulthood	9/10 Validity ✓ Clear aims ✓ Appropriate qualitative methodology ✓ Appropriate research design ✓ Appropriate recruitment strategy ✓ Appropriate data collection ✓ Considered reflexivity appropriately Results x Ethical considerations addressed ✓ Rigorous data analysis ✓ Clear statement of findings Utility of results ✓ Value of research
Lachal et al. 2012 [47]	5 (3 ♂)	Aged 14–17. Receiving therapy for past depressive episode, 4 participants use medication. Selected after consultation in psychiatry department. Episode was over at the time of interviewing (reflection).	France	How a qualitative method, using in-depth interviews with patients and clinicians, can help building a specific tool to measure depression in adolescents.	Semi-structured interviews	Thematic Analysis	1) Emotional state 2) Non-emotional manifestations 3) Manifestations in social interactions	6/10 Validity ✓ Clear aims ✓ Appropriate qualitative methodology x Appropriate research design ✓ Appropriate recruitment strategy x Appropriate data collection x Considered reflexivity appropriately Results ✓ Ethical considerations addressed x Rigorous data analysis ✓ Clear statement of findings Utility of results ✓ Value of research
McCann et al. 2012 [48]	26 (15 ♂)	Aged 16–25. Purposeful sampling at Headspace via clinicians 1) depression as primary diagnose 2). Excluding psychosis and suicidality. 13 had double diagnose depression + anxiety	Australia	Examine the lived experience of young people diagnosed with depression	Semi-structured interviews	Interpretative Phenomenological Analysis	1) Struggling to make sense of their situation 2) Spiralling down 3) Withdrawing 4) Contemplating self-harm or suicide	8/10 Validity ✓ Clear aims ✓ Appropriate qualitative methodology ✓ Appropriate research design x Appropriate recruitment strategy ✓ Appropriate data collection x Considered reflexivity appropriately Results ✓ Ethical considerations addressed ✓ Rigorous data analysis ✓ Clear statement of findings Utility of results ✓ Value of research
Meadus 2007 [49]	9 (8 ♂)	Aged 15–18 diagnosed with mood disorder (7 depression, 2 bipolar). Treated by psychiatrist or GP. Receiving medication. Both inpatient and outpatient.	Canada	Explore the phenomenon of coping as experienced by adolescents with a mood disorder	Unstructured interviews	Grounded theory, each interview analysed before starting the next interview	1) Feeling different 2) Cutting off connections 3) Facing the challenge /reconnecting 4) Learning from the experience	7/10 Validity ✓ Clear aims ✓ Appropriate qualitative methodology ✓ Appropriate research design x Appropriate recruitment strategy ✓ Appropriate data collection x Considered reflexivity appropriately Results ✓ Ethical considerations addressed x Rigorous data analysis ✓ Clear statement of findings Utility of results ✓ Value of research
Midgley et al. 2015 [50]	77 (55 ♂)	Aged 11–17. Diagnosed with depression with moderate to severe impairment. Part of larger RCT (IMPACT study) clinically referred to child and adolescent mental health services for treatment for depression, but have yet to receive therapy	UK	Exploring the experience of depression in a sample of young people aged between 11 and 17	Semi-structured interviews (before intervention, rather brief 4-37 min)	Framework analysis	1) Misery, despair and tears 2) Anger and violence towards self and others 3) A bleak view of everything 4) Isolation and cutting off from the world 5) The impact on education	9/10 Validity ✓ Clear aims ✓ Appropriate qualitative methodology ✓ Appropriate research design ✓ Appropriate recruitment strategy xAppropriate data collection ✓ Considered reflexivity appropriately Results ✓ Ethical considerations addressed ✓ Rigorous data analysis ✓ Clear statement of findings Utility of results ✓ Value of research
Rosales 2008 [51] (PhD thesis)	6 (5 ♂)	Aged 12–15 and diagnosed with bipolar disorder, dysthymia, and major depression. Purposeful sampling through community counsellors and school districts professional counsellors, private therapists, school psychologists. Students included were required to have a support system in place such as parents and peers.	USA	middle school adolescents their thoughts and perceptions about their experiences with depression	Semi-structured & open-ended	Comparative method analysis	1) Person centred 2) Hopelessness 3) Relationships/ connections 4) isolation 5) Escape/ distractions	7/10 Validity ✓ Clear aims ✓ Appropriate qualitative methodology ✓ Appropriate research design xAppropriate recruitment strategy ✓ Appropriate data collection ✓ Considered reflexivity appropriately Results x Ethical considerations addressed x Rigorous data analysis ✓ Clear statement of findings Utility of results ✓ Value of research
Weitkamp et al. 2016 [52]	6 (5 ♂)	Aged 14–19. Interviewed after max two sessions with therapist suffering from depressive disorder. Exclusion criteria: cognitively too impaired to participate as rated by therapist/interviewer. All met criteria depression ICD and some with comorbidity of PTSS, anxiety and bereavement.	Germany	Lived experience of young people diagnosed with depression, and additionally to look at the way these YP accessed therapy in the context of the German mental health system.	Semi-structured interviews	Interpretative Phenomenological analysis	1) Suffering is experienced as overwhelming 2) An experience of loneliness and isolation 3) Struggling to understand the suffering 4) Therapy as a last resort	8/10 Validity ✓ Clear aims ✓ Appropriate qualitative methodology ✓ Appropriate research design x Appropriate recruitment strategy ✓ Appropriate data collection ✓ Considered reflexivity appropriately Results x Ethical considerations addressed ✓ Rigorous data analysis ✓ Clear statement of findings Utility of results ✓ Value of research
Woodgate et al. 2006 [53]	14 (11 ♂)	Aged 13–19. Outpatient diagnosed with depression for > 18 months. 12 other mental health condition as well (e.g. ADHD/substance abuse) but no other severe mental illness.	Canada	Gain an understanding of what it was like to be an adolescent living with depression.	Open-ended interviews & Focus groups (same participants in groups of 6)	Hermeneutic phenomenology	1) Containing the shadow of fear 2) Keeping the self alive 3) Maintaining a sense of belonging in the world 4) Feeling valued as a human being	6/10 Validity ✓ Clear aims ✓ Appropriate qualitative methodology ✓ Appropriate research design x Appropriate recruitment strategy ✓ Appropriate data collection x Considered reflexivity appropriately Results ✓ Ethical considerations addressed x Rigorous data analysis x Clear statement of findings Utility of results ✓ Value of research

### Data analysis

For each included study, one researcher (LA) identified any text relating to loneliness in the results section (whether quotes or interpretation) and imported relevant passages into a qualitative data analysis software package [54] to facilitate the process of thematic synthesis. Three researchers (AP, MB, EP) independently assessed a set of studies each to identify which passages to import and compare judgements on which data to include or exclude.

Having established our final database of extracted qualitative data, one researcher (LA) then coded the full dataset, and three researchers (AP, MB, EP) independently coded data from two randomly-allocated studies each. All four researchers then compared their coding to develop an initial coding framework. This was then refined through an iterative process, to develop a taxonomy of analytical themes.

### External validity

To improve external validity and reduce researcher subjectivity, we presented the findings of this draft thematic framework at an inter-disciplinary research workshop held in London on 26th June 2019. This formed part of the research activities of the United Kingdom Research and Innovation (UKRI) Loneliness and Social Isolation in Mental Health Research Network (see Acknowledgements). The 58 participants included health and social care practitioners, university and voluntary sector researchers, policy makers, lived experience researchers, and mental health service users. Following an oral presentation of findings, one researcher (LA) led two independent 45-min sessions with 8 attendees in each group to discuss the coding framework in more detail. The comments made were used to revise the coding framework and improve reflexivity.

### Reflexivity

Our multidisciplinary research team reduced the dominance of one perspective. This was important, as a meta-synthesis is an overarching interpretation from the joint analysis of primary studies [33], with a high risk of subjectivity and personal bias. LA is a social scientist and medical student with an interest in the links between mental health and loneliness, AP and SJ are psychiatrists and academics with an interest in sociology and social psychology, EP has a research background in experimental psychology and biological anthropology, with experience of having worked in the voluntary sector, while MB is a mental health occupational therapist and academic. This team approach, and the use of a multidisciplinary research workshop to discuss findings, reduced the focus on loneliness from a medical perspective by including insights from multiple disciplines.

## Results

### Description of included studies

**Fig. 1:**
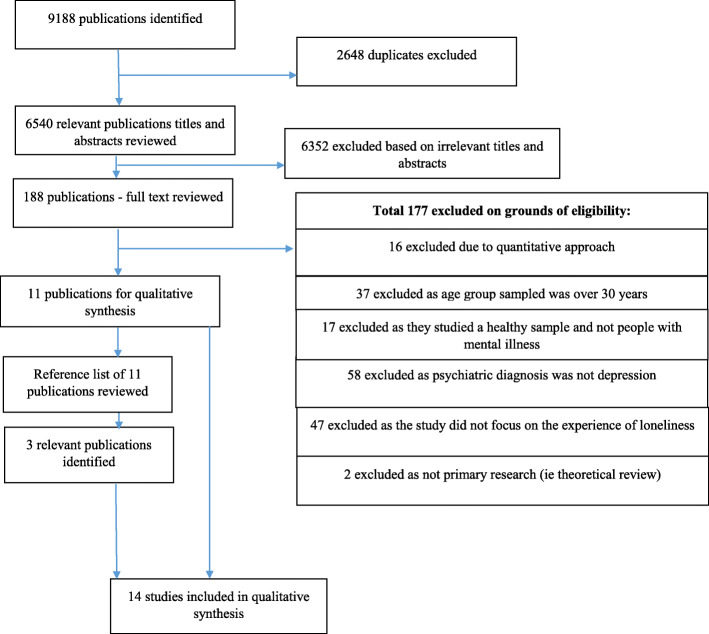
Flow diagram for included studies

Our search identified 9188 studies, which was reduced to 6540 after deleting 2648 duplicates (see Fig. 1). Following screening of titles/abstracts we excluded 6351 studies for irrelevance. Following full text review of the remaining 188 studies we excluded 177 based on our exclusion criteria, included eleven studies and added three more based on hand searching the references from the selected studies, identifying 14 eligible studies [24, 41–53], which we included in this meta-synthesis. We achieved 100% agreement on study eligibility between four authors. Characteristics of each study are shown in Table 1, including an assessment of study quality using CASP criteria.

The total number of participants was 388, with sample sizes in each study ranging from 5 to 107. Participants’ ages ranged from 11 to 30 years, and roughly three quarters were female (*n* = 288). Dates of publication ranged from 2002 to 2019, and studies originated from the United States, United Kingdom, Finland, Belgium, France, Canada, Australia, and Germany. Thirteen studies were published in peer-reviewed journals, while one study was an unpublished PhD dissertation [51]. One study was a meta-synthesis of six qualitative studies [43], which included one identified in our own search [44]. We decided to include this meta-synthesis as a unified whole rather than disaggregating its included studies because a number of those studies were unpublished theses that were unavailable to us.

All studies involved individuals with a history of depression, whilst two studies included participants with depressive episodes in the context of bipolar disorder. In those two studies an unknown proportion had bipolar in one [51], whilst two out of nine participants had bipolar in the other [49]. Most studies (*n* = 12) collected interview data, while two studies used free text from written self-reports. Included studies used a range of qualitative analytic methods: thematic analysis, interpretative phenomenological analysis, grounded theory, discourse analysis, framework analysis, hermeneutic phenomenology, content analysis, and comparative method analysis.

### Thematic synthesis

Our thematic synthesis of 14 eligible studies identified four analytic themes: (1) social withdrawal due to poor mental health, (2) non-disclosure of depression contributing to social distance (with four sub-themes), (3) the desire to connect, and (4) paradoxes of loneliness and depression. Quotes given in italics denote primary data.

### Theme 1: social withdrawal due to poor mental health

A key theme we identified related to the debilitating nature of depressive symptoms, which made it very hard for some young people to engage with others. Nearly all studies (*n* = 13) described the experience of depression as causing those individuals to withdraw from others, relating this to difficulties in being around others due to low motivation and low energy.

*“There would be days that I just couldn’t get out of bed. I didn’t want to face people. I didn’t want to look at anybody, I just wanted to stay there and I guess just sulk by myself, and I just didn’t have any energy.”* (Female in her 10s, USA sample) [43].

Some individuals described feeling better when isolating themselves from peers, because being around others was so emotionally draining*. “I come home it’s just kind of like a relief”*, explained Lana (teenager, UK sample), who had been bullied at school for reasons unspecified [50]. Some participants avoided others by spending time in their rooms or going for walks alone. One female participant in her teens from the USA explained*, “I just kind of wanted to be by myself.”* [41]

Participants described having stopped participating in activities they had previously enjoyed or not feeling able to fully engage in such activities. A female in her teens from the USA, who had taken an active role in the performing arts since the age of 2 years, explained.

*“I was in show choir and throughout that year I just didn’t really enjoy it. I was fine with standing in the back, which really wasn’t like me. My wanting to be in the back just wasn’t normal.”* [41]

Low self-esteem seemed to affect some young people sampled, who felt that their depression had worsened their insecurities, leading them to withdraw socially. The depression had apparently eroded their belief that anyone could find them likeable, resulting in them withdrawing to avoid other people.

*“I become even more withdrawn than I normally am, and it’s based on the insecurity, and it came up the unlikeability thing again, that I’m not likable inherently so what’s the use of pretending that I am because eventually they are going to find out.”* (Sarah, teenager; Canadian sample) [45].

Participants also spoke of an inability to feel affection from others: “*When you’re depressed you feel like you don’t have anybody.*” (Tina, teenager, USA sample) [44]. The syndrome of depression set young people apart from their peers and made them feel different. This change was noted by others, even if they did not necessarily recognise it as depression, and this could lead to others’ withdrawal. The sense of rejection was apparent in young people who coped by isolating themselves, thus compounding their sense of differences between them and others.

*People just started drifting away, like they were asking, “What’s wrong with you?” I wanted to ask them, “Why don’t you talk to me anymore?” I felt they were saying “You’re different now!” I just began to hide away a lot and I would say, “I just want to be alone”.* (female teenager, USA sample) [51].

### Theme 2: non-disclosure of depression and social distance

The second theme, emerging from 12 studies, was more explicitly related to feelings of loneliness. As young people dealing with depression were hesitant about disclosing their depressed feelings to people in their social networks, they avoided being open about their true selves. This sense of otherness through concealment enhanced participants’ feelings of loneliness. Some individuals described being very aware of putting up a façade and of making extensive efforts to maintain this front to avoid talking about their mental health issues.

“*I would put on a smile for my parents and my siblings. Whenever somebody would leave and I knew I was going to be alone, they would ask me, “Are you going to be alright?” And I would say “Yes, of course,” because I didn’t want them to know what I was dealing with. But, it was a living hell. I put up a really good façade for them, like all cheery and happy, nothing’s wrong.”* (Female in her 20s; USA sample) [41].

A range of reasons were given for the non-disclosure of depressed mood, summarised in the four sub-themes below.

### Subtheme 2.1 fear of being judged

Young people in the included studies commonly expressed fear of being judged negatively if they identified themselves as suffering from depression, or of being perceived as unbearable or embarrassing if they vented their feelings. The negative consequences they feared included social exclusion and isolation, as borne out by their experiences:

“*I cannot talk about my sadness, in fact, I don’t dare to talk about it, because then you are considered as a weak person. I see that some people feel pity for me, but they don’t talk to me, they prefer to run away because they are afraid and do not know how to react to someone who is sad.”* (Female, teenager, Belgian sample) [24].

“*If I could talk to them [friends] I would, but I just didn’t feel like I could talk to them. They would keep on going, ‘You’re weird’ or something.” (Sandra, teenager, USA sample)* [44].

### Subtheme 2.2 preserving friendships

Another reason for not disclosing their depression was that the young people sampled clearly valued their friendships and wanted to preserve existing networks. They feared losing these connections if they shared their feelings of depression. There was also a fear of burdening others, in that by not disclosing their depressive thoughts they hoped to minimise the negative impact of their depression on others. Many adolescents had experienced negative changes or the ending of friendships as a consequence of mental health problems and this reinforced their reluctance to reveal their feelings to friends.

*“I’m afraid that friends and significant others can’t see me the same way as before or something might change between us if I told them all my troubles. I don’t want to bother anybody with my worries.” (*Unknown gender, 15–17 years old; Finnish sample) [42].

### Subtheme 2.3 difficulty explaining oneself

Beyond deliberate efforts to avoid talking about their feelings, young people also found it hard to explain why they felt depressed. Pressure to explain themselves arose from members of their peer group, who struggled to comprehend their experiences, expressing this through intolerance. Their own inability to formulate or articulate an explanation frustrated young people with depression and had the effect of widening the gulf between them and others.

“When you feel bad, you need to have an external explanation for why you have these feelings, because the fact that you feel bad must be caused by something. Participants stated that they often received the question: ‘*Why are you feeling so bad?*’ Adolescents shared that they cannot give a constructive answer because they do not know why they have these feelings. They could not give explanations because there were no specific causes for them. Due to the inability to provide a real explanation regarding the causes, their feelings and depression are not recognized by others.” [24]

### Subtheme 2.4 perceived futility of explaining oneself

Experiencing depression engendered a realisation of being different from one’s peers. This gave rise to the belief that others would not understand one’s situation and that there was therefore no point in discussing it. Young people with depression indicated that they feared others were likely to trivialise, dismiss or ignore their depressive symptoms. Again, their previous negative experiences of others failing to understand them taught some young people not to disclose their feelings. The lack of any incentive or opportunity to confide and feel understood made young people feel very lonely.

“Having others reach out, however, was not always beneficial. Some participants, especially females, did not feel comfortable opening up to those who reached out to them. These participants did not believe the other person would understand what they were going through, believed their problems were ‘*no one else’s business*’ or doubted the person’s motives for reaching out.”

[41]

“Despite the fact that all the individuals in this sample acknowledged social support as an important part of their daily lives, the belief that others cannot understand their experiences often caused individuals to feel alone.” [46]

### Theme 3: the desire to connect

Despite young people reporting a tendency to disengage from certain social interactions, they still expressed a desire for connection and a desire to feel ‘normal’.

“At the same time, the adolescents hoped to have more friends and to be included in their peer group. In addition, they wished to have a good time with the friends and to have somebody to talk to about their problems and feelings.” [42]

“Most individuals have a strong need to connect and have positive relationships with others especially middle school students.” [51]

In this sub-theme we identified a conflict with the experiences described in sub-themes 2.3 and 2.4 above, in that although some individuals expressed a wish to talk about their issues, they also experienced difficulties in doing so. Such barriers included a fear of the consequences, particularly the threat of rejection (and perhaps stigmatisation) from peers. To address this, some preferred to share their problems with people who they knew had faced the same mental health issues in preference to their wider peer group, amongst whom it was not always clear who had experienced depression themselves.

Shadow clearly had the wish to disclose to someone, which he expressed in a wish for some kind of group therapy to meet people where he could actually speak about his problems: “*And maybe, that you can talk about it in a group that you can say: “I am [Shadow], I have this and that problem. What do you think, what is your impression, what is your problem?” . .*. *Because I can’t possibly walk into my classroom and say: “you know what happened to me?” Well, I could, but*. *.*.” (Male, teenager; German sample) [52].

### Theme 4: paradoxes of loneliness and depression

This theme described a number of paradoxes or vicious cycles that were apparent in various forms across a number of studies. Whilst some young people talked about a need or a tendency to withdraw socially, this came with an awareness that such avoidance could create or worsen feelings of loneliness.

“During their depressive experiences, participants felt a distinct separateness from others and often chose solitude over being with others even when feeling lonely” [44].

*“Being around people was, was always a bad thing for me. I constantly felt the need to be alone*. *.*. *and I always felt like interacting with other people was difficult for me*. *.*. *Ya, that was confusing because I felt lonely but I didn’t feel like being around anyone at the same time”* (Jeff, in his 20s, Canadian sample) [45].

Some young people described their friends showing a form of understanding by not asking too many questions, but then feeling cut off because of an apparent absence of overt concern.

*Sometimes when some of my friends are … .. ok with ignoring me, with not asking about it, I feel like kind of I know it’s ridiculous, but unloved.* (Female, teenager, UK sample) [50].

Another trap that some young people described was a vicious cycle of loneliness and depression, was the suggestion that the manner in which they processed feelings about loneliness reinforced their depression.

“They were unable to initiate or sustain relationships because of feelings of severe discomfort around people. They described a cycle of feeling lonely, often as a result of their breakups, and then feeling depressed about the loneliness, causing a self-fulfilling prophecy by further alienating and self-isolating themselves from others.” [45]

A fear of stigma was also mentioned as a reason for withdrawing from others, but this came at the price of increasing loneliness. Sometimes a yearning to connect with others coexisted with an inability to be with them. However, where they withdrew from others, young people were prevented from getting support from others, thus increasing their sense of alienation from friends.

“While some disclosed their depression to friends, others withdrew, fearful of the perceived stigma and loss of status from being labelled as having mental illness …. However, retreating from others contributed to their loneliness and isolation.” [48]

The difficult choice that some young people faced, was between withdrawing socially to hide their depression and then feeling excluded, or remaining superficially socially engaged but living behind a façade in not disclosing their depression. In the latter case, the strain of concealing their low mood could create a sense of greater alienation from their peers.

“This process of social isolation was characterized by ambivalent feelings. Participants explained that on the one hand they feel the necessity to share their emotions with others, but on the other hand they felt it was impossible to do this. Consequently, they felt caught up in a vicious circle which made them feel alienated from themselves and from of their social world.” [24].

No suggestions were made by participants as to how to break such vicious cycles, but a note of optimism was sounded in relation to recovery from depression. During an episode of depression young people characteristically described the experience of yearning for a connection with others, feelings of being very different from others, and a perception that their problems were incomprehensible to their peers; all of which meant that when unwell, gaining a sense of connectedness was out of their reach. However, on recovering from an episode of depression, those who reflected back on those unwell periods had better insight into such traps and were able to see how a lifting of their symptoms removed many of the barriers to connecting with others.

## Discussion

### Main findings

This meta-synthesis of fourteen qualitative studies describing the experience of loneliness among young people with depression identified four main themes, conveying the social consequences of both loneliness and depression, and illustrating a range of pathways between them and a sense of how these could be mutually reinforcing. Young people described the symptoms of their depression as leading to social withdrawal. Although they did not name them as such, the symptoms they described match those featuring in diagnostic criteria for depression (low energy, anhedonia, avolition, low self-esteem). The first theme suggests that although debilitating, these symptoms could lead to social isolation but not necessarily feelings of loneliness. Indeed, some young people gained a sense of relief when not having to engage in their usual social roles although this pattern of social withdrawal risked increasing the probability of feeling lonely over the longer term. Our second theme was more explicitly related to loneliness, capturing how non-disclosure of depression made it hard for young people to feel connected because it distanced them from others. Contrary to the experiences of those who preferred to avoid others even at the cost of feeling lonely, data coded under our third theme illustrated a longing to be among others. Our fourth theme described a set of paradoxes faced by young depressed people, including that of yearning for connection, or believing it might help with low mood, yet being unable to tolerate being around others. This theme also described a self-perpetuating cycle of loneliness and depression, providing insights into the cognitive processes underlying this bidirectional relationship. Our data suggest that where depressed individuals engage in certain behaviours (withdrawing and not confiding) this can lead to feelings of loneliness, an awareness of which worsens their mood, thus perpetuating their depression. Again, this process of self-isolation and social alienation builds a sense of one pathway in an apparent bidirectional relationship.

We did not observe any gender or age patterning of themes, but it was hard to rule these out without access to the primary data. Ranging in age from 11 to 30 years, many older subjects reflected back on their previous experiences, but it was not always clear what age they were referring to. We also did not identify any mention of difficulties accessing social contacts or in meeting people, in contrast to the issue of sparse social networks being described as a problem for older age groups [55, 56]. In our data psychological aspects of depression were more prominent than social aspects of depression. Although some participants mentioned social anhedonia there was little mention of impaired social communication (for example impaired emotion recognition) or of impaired social perception (for example reduced empathy), as might otherwise be seen in depression [57]. However, participants may have lacked an awareness of this and we lacked collateral accounts to triangulate their own. We also did not identify overt descriptions of perceived stigma, although this was implied in an avoidance of disclosing depression to peers. Our themes built up a theoretical framework focussed on a dominant pathway of depression leading to loneliness (and loneliness worsening depression) than on loneliness having predated (and/or contributed) to the onset of depression. However, the nature of our study meant that it was impossible to probe individuals’ thoughts about predisposing factors and this warrants further interview work.

### Findings in the context of other studies

We believe this to be the first meta-synthesis of qualitative studies describing the experience of loneliness in young people with depression. Quantitative testing of causal models of adolescent depression suggest that loneliness and low self-esteem increase the probability of depression, with low self-esteem having an indirect effect on depression via loneliness [58]. Our data implied that loneliness was not an antecedent of depression so much as a consequence of it, although none of the included studies probed this. Whilst we did not identify any gender patterning, US survey data suggest that loneliness was one of the most common self-reported features of depression among female adolescents with depression but far less apparent among depressed boys [16].

Our finding that some young people distance themselves from others through a fear of being rejected is consistent with the interpersonal hostility theory of loneliness [30]. This theory posits that loneliness generates negative social cognitions through which others can be perceived as threatening, competitive and unwelcoming. This leads to a self-fulfilling prophecy in which social contact is put on hold through fear of negative evaluation [30]. This process of interpersonal hostility leads to more isolation, which increases the likelihood of feeling lonely, creating a self-reinforcing loneliness cycle accompanied by feelings of low self-esteem [59].

Empirical studies exploring the responses of young people towards peers suffering from mental illness demonstrate clear stigmatising attitudes towards people with mental health problems and a preference to avoid them [60]. This would confirm what young people with depression fear: being judged and avoided. Their preference for non-disclosure is therefore unsurprising. Such work also shows that young people’s attitudes towards peers with mental health problems are influenced by their parents’ attitudes and by their previous exposure to people with mental health problems [60]. This suggests that there is scope to modify attitudes and create more accepting environments for young people with depression.

Adolescence is a time characterized by hormonal, physical, psychological and social change [61], during which identity formation, role transition, independence and creating relationships are of critical importance [62]. During this period adolescents become less dependent on parental attachment as peer relationships become more important [63], but this can mean that the effects of social exclusion by peers are felt more acutely [3, 63]. Attachment theory suggests that psychiatric symptoms (such as depression) and feelings of loneliness arise when there is an absence of opportunity to make affectional bonds, or when bonds once made are repeatedly disrupted [64]. It is also theorised that young people with attachment disorders come to define themselves as outsiders and through this they risk becoming chronically lonely [62]. It is possible that early experience of unreliable and unresponsive attachment figures can lead to insecure attachment perceptions, including a lack of trust, low self-esteem and difficulties with affect regulation and intimacy [63]. This is supported by evidence that lonely young adults are more likely to have been bullied and socially isolated as children [17] and that securely attached adolescents report more emotionally close friendships and greater social acceptance by peers than insecurely attached peers [63]. Such work suggests that early parenting interventions to improve attachment have the potential to prevent loneliness and depression [65], although this requires formal testing.

### Strengths and limitations

This meta-synthesis used a robust systematic search strategy to identify studies collecting qualitative accounts of loneliness in young people with depression across a range of countries. We followed received guidelines on conducting a meta-synthesis and used an interdisciplinary team approach in conducting our analysis. We addressed threats to validity by presenting preliminary results at a workshop attended by academics, voluntary sector practitioners, and people with lived experience, requesting feedback with which to revise our thematic framework.

The nature of our research question meant that research subjects were hard to reach and it is possible that those willing to participate in included studies are not representative of the wider population of depressed young people. The predominance of female participants from high-income countries, also limits generalisability. Whilst our research question related specifically to the experience of loneliness in the context of depression, our search strategy did not restrict eligibility to studies that had similar aims. Instead, by including studies with the broader remit of understanding the experiences of depression among young people we were able to identify instances where loneliness was mentioned in this context. Whilst this meant that included studies did not necessarily probe the experience of loneliness in depression, restricting the richness and variety in data relevant to our research question, we were able to identify and describe this where it was mentioned. Any meta-synthesis analyses the findings of previously analysed data and is therefore a subjective interpretation of an interpretation [34]. This could potentially undermine the integrity of individual studies by ignoring the context, leading to superficial interpretations rather than a deeper understanding [36]. This was a particular threat in our meta-synthesis given the range of analytic approaches used in the constituent fourteen studies. A further limitation of the meta-synthesis approach is that such interpretations are made by a group of researchers with their own perspectives, which might plausibly differ from those of another team. However, we addressed this through our consideration of reflexivity in discussions of the multidisciplinary research team. To address some of these limitations and gain a more in-depth understanding of the pathways between loneliness and depression in young adults, it would be important to conduct qualitative interview studies with a specific focus on these links.

### Clinical and policy implications

Our study provides valuable insights for clinicians, teachers, parents, peers and researchers into the social challenges faced by young people with depression, helping them understand how feelings of loneliness might arise in those who feel depressed and how they might compound depressive symptoms, impeding recovery. This is consistent with the evidence that poor subjective social support is associated with poorer recovery from depression [10].

It was common for research participants to feel that others did not understand them and the distress associated with this was very apparent. Lay dissemination of the findings of this study might help young depressed people feel more understood by those in their social networks and suggest ways in which others can support them appropriately. There is evidence that social media campaigns focussed on mental health awareness and stigma reduction can promote help-seeking for mental illness, but this is thought to be attributable to wider societal awareness in creating environments where it is acceptable to disclose mental illness [66]. Creating a culture in which it is more acceptable to disclose mental ill-health has the potential to interrupt the vicious cycle of social withdrawal giving rise to loneliness and thereby worsening mood. It may be useful for clinicians to explain to young people with depression that it is common for young patients not to disclose mental health difficulties to their peers, but that this might potentially reduce their sense of connectedness. Clinicians might be able to educate them about the impact of loneliness on the prognosis of their depression and explore appropriate ways to address this.

Early intervention in loneliness would appear to be critical to prevent lonely young adults from being trapped in loneliness as they grow older [17] and to prevent it from limiting psychosocial functioning and diminishing quality of life at an important stage of social and emotional development. However, whilst this study provides an understanding of the links between loneliness and depression in social terms, it does not detail the psychological processes involved in those pathways. A better understanding of the psychological factors that engender and perpetuate loneliness in young people with depression would help identify those that are modifiable and contribute to developing effective interventions. Combining insights from cognitive neuroscience and social psychology would be helpful in the foundations for this interventional work.

### Future research

We have mentioned the need to conduct interview studies directly probing the experience of loneliness in young people with depression, with balanced gender representation. Given the lack of ethnic diversity in our sample and cultural dimensions of social connections, we also need studies conducted in different ethnic groups. Such work might probe, for example, the more extreme phenomenon of hikikomori in Japan where young people with mental health problems engage in extreme self-isolation [67]. We have also mentioned the need to develop effective interventions. In the wider literature on approaches to addressing loneliness among people with mental health problems, changing cognitions to shift maladaptive cognitions is viewed as most promising, but lacks a robust evidence base [68]. Amongst trials of interventions to improve subjective and/or objective social isolation for people with mental health problems, again the most promising interventions include cognitive modification for subjective social isolation, as well as interventions with mixed strategies and supported socialisation for objective social isolation [69]. More research is needed to develop and assess the acceptability of interventions that address cognitions about social engagement among young people with mental health problems, before trialling them rigorously.

## Conclusions

Our meta-synthesis of fourteen qualitative studies capturing experiences of loneliness among young people with depression identified four themes revealing the challenges faced by these individuals in their social networks. They described how the symptoms of their depression hampered social engagement, leading to social withdrawal. A preference not to disclose their mental health problems, for a variety of reasons, compounded the perception of differences between depressed young people and their peers. Many longed for a connection with others, but could not tolerate the experience of being with others. Some participants described a self-reinforcing cycle of loneliness and depression, from which it was hard to see a way out. Although participants did not suggest how to intervene to break this cycle, our findings suggest that supporting young people with depression to find appropriate ways to disclose their problems has potential to promote a sense of feeling understood by their peers. The published literature also suggests a role for cognitive interventions to shift maladaptive cognitions about their social world. More widely, a change in societal attitudes towards young people with mental illness would also help promote a sense of feeling accepted and socially connected.

## Data Availability

All data are published and in the public domain.

## Appendix 1

### Search strategy

To reduce the risk of missing relevant studies our initial search protocol included a term to capture a diagnosis of personality disorder. However, as per our inclusion/exclusion criteria, only studies relating to young people with depression were included.

MeSH terms:

Mood disorder / Affective disorder / Personality disorder / Anxiety disorder /

(and related relevant MeSH depending on database)

OR Depress*

OR Personality disorder**

OR Borderline personality disorder or emotionally-unstable personality disorder or histrionic personality disorder or narcissistic personality disorder

OR Avoidant personality disorder or dependent personality disorder or obsessive/compulsive personality disorder)

**AND Loneliness**

loneliness [MeSH] OR loneliness OR lonely

(social* adj/2

isolation

network*

support

contact*

relation*

capital

distance

alienat*

detach*

tie*

participation

activ*

engage*

connect*

disconnect*

cohes*

embedded*

interact*)

**AND Qualitative research**

“Qualitative research”

Interviews as Topic

“Qualitative adj2 (stud* OR method*)”

“Lived experience”

“Mixed?method*”

“Thematic analysis”

“semi-structured” or semistructured or unstructured

(guide*) adj2 (interview* or discussion*))

Focus groups

ethnograph*

fieldwork

group discussion

Patient perspective

**NOT (in title)**

cancer

HIV

AIDS

Parkinson*

Alzheimer*

Mild cognitive impairment

Brain injury

Kidney failure

Renal disease

hemodialysis

Diabetes mellitus

Cystic fibrosis

Coronary heart

caregiver*

Carer

**

We ensured that personality disorder was well-represented in our search terms so that we could conduct two meta-syntheses of qualitative studies with young people based on this search. This article relates only to studies in which the primary diagnosis was depression.

## Abbreviations

AMC: Academisch Medisch Centrum (Academic Medical Centre)
CASP: Critical Appraisal Skills Programme
UCL: University College London
WHO: World Health Organisation
UK: United Kingdom
UKRI: United Kingdom Research and Innovation
UMC: University Medical Centre
USA: United States of America
